# Acceptability and usability of a respiratory biosensor for drug overdose detection and first responder notification: a qualitative evaluation of perspectives from people who use drugs and wider stakeholders

**DOI:** 10.1186/s12954-026-01452-8

**Published:** 2026-05-21

**Authors:** Kristina Hnízdilová, Andrew Radley, Madeleine Caven, Brian Paul Stephens, Christopher J. Byrne, Osian Meredith, Bruce Henderson, John Francis Dillon

**Affiliations:** 1 Division of Respiratory Medicine and Gastroenterology, University of Dundee, Dundee, Scotland; 2 Division of Population Health and Genomics, University of Dundee, Dundee, Scotland; 3 School of Medicine, University of St Andrews, Fife, Scotland; 4 Department of Gastroenterology, Ninewells Hospital and Medical School, NHS Tayside, Dundee, Scotland; 5 PneumoWave Limited, Glasgow, Scotland

**Keywords:** Respiratory biosensor, Digital health, Process evaluation, Harm reduction, Opioid-induced respiratory depression, Qualitative research, Observational cohort study, Dundee, Scotland, United Kingdom

## Abstract

**Background and aims:**

Overdose induced by illicit drug use is a significant public health threat in many settings globally. Many such overdoses occur in the context of lone use in private spaces, in the absence of potential first responders. Chest-worn biosensors have the potential to detect respiratory depression which signal overdose and alert first responders, enabling rapid reversal. However, the acceptability of this approach to people who use drugs and other key stakeholders are not known. We aimed to investigate these outcomes in this qualitative sub-study, which is part of a larger trial investigating this intervention.

**Methods:**

Semi-structured interviews and focus groups were conducted with participants who completed the study protocol (*n* = 20), partially completed the study protocol (*n* = 1) and stakeholder groups (*n* = 8) about device acceptability. Verbatim transcripts were analysed using reflexive thematic analysis. Factors influencing device acceptability for PWUD were interpreted using the concepts of the COM-B behaviour model. Discussions around implementation with stakeholder groups utilised Normalisation Process Theory to further appraise the intervention and its integration into existing services.

**Results:**

Experiences with overdose or drug-related deaths (DRD) were identified within qualitative data as motivating factors for device wear. First responder groups stressed the importance of patient choice and device accuracy. The accelerometer sensor was found to be acceptable to people who use drugs (PWUD) and was utilised to monitor respiratory patterns. Stakeholders recognised the potential of these devices to play a part in the management of overdose and identified necessary requirements to ensure successful implementation of the device, such as funding allocation, potential barriers and integration into existing services.

**Conclusions:**

The chest-worn biosensor was acceptable to PWUD, driven by personal experiences with overdose risks, and were viewed by stakeholders as a promising tool for overdose management. Successful implementation will require ensuring device accuracy, respecting patient choice, securing funding, and integrating the technology into existing services.

**Clinical trials registration:**

Researchregistry7351

**Electronic supplementary material:**

The online version of this article (10.1186/s12954-026-01452-8) contains supplementary material, which is available to authorized users.

## Introduction

In recent years, drug related deaths (DRD) have been one of the leading causes of death among vulnerable people [1]. The contributory factors are numerous, such as longstanding social deprivation, adverse childhood experiences and lack of investment in social services.

In Europe, Scotland experiences the highest per-capita DRD rates, peaking in 2020 when Scotland experienced the highest DRD rate since records began. Dundee, Scotland’s fourth largest city, where this study was conducted, has consistently recorded the highest drug-related death rates in the country. The main drugs contributing to DRDs in Scotland are opioids and non-prescription benzodiazepines with the majority of DRDs caused by opioid-induced respiratory depression. Lone drug use has contributed to these statistics. In 2020, 68% of DRDs in Tayside, Dundee’s health board region, had occurred when individuals used drugs alone [2].

Supervised consumption rooms (SCRs), where illicit drugs can be used under supervision, are associated with lower overdose mortality [3] By 2023, sanctioned SCRs were established in Canada and Australia. In Vancouver, SCR attendance is associated with increased rates of referral to drug rehabilitation treatment and opioid agonist therapy (OAT) [4, 5]. SCRs have not been introduced in the United Kingdom until recently, due to drug legislation and political opposition [6]. The UK Government had cited the lack of established trial data on the efficacy of SCRs, which is difficult to collect due to many confounders. The Scottish Government has recently published an evidence paper about the benefits of SCRs and a pilot SCR opened in Glasgow in January 2025 with funding allocated for its costs from 2024 to 2027 and a further SCR proposed to open in Edinburgh [7, 8].

Wearable devices, such as the device used in this evaluation, offer the opportunity for a portable and discreet digital safety net during drug use, for those who cannot, or choose not to, access SCRs, with development progressing rapidly [9, 10]. Furthermore, these devices may offer a solution in areas where SCRs are currently unavailable due to legislation or funding issues and PWUD who do not want to use SCRs.

This paper presents the findings of a qualitative sub-study of a trial examining the feasibility of a respiratory biosensor to detect and alert overdose among PWUD. While the trial is examining respiratory patterns and associated outcomes, this study evaluates the acceptability of the sensor to PWUD, healthcare staff, and stakeholders; and barriers and facilitators to its use by PWUD. The study also explores contextual factors influencing integration of the device into an intervention pathway to reduce DRDs.

## Methodology

This study obtained regulatory approvals (University of Dundee sponsorship, NHS East of Scotland Research Ethics Committee, Research & Development NHS Tayside) by January 2022, prior to the beginning of the full RESCU study (REC 21/SS/074, IRAS 301153, study registered on Research Registry on November 10, 2021, researchregistry7351). The COREQ 32-item checklist was used to report study findings [11].

### Study design

This is a qualitative study to assess participant and stakeholder responses to experiences of PWUD using the device to record respiratory patterns.

### Respiratory device and intervention description

**Fig. 1:**
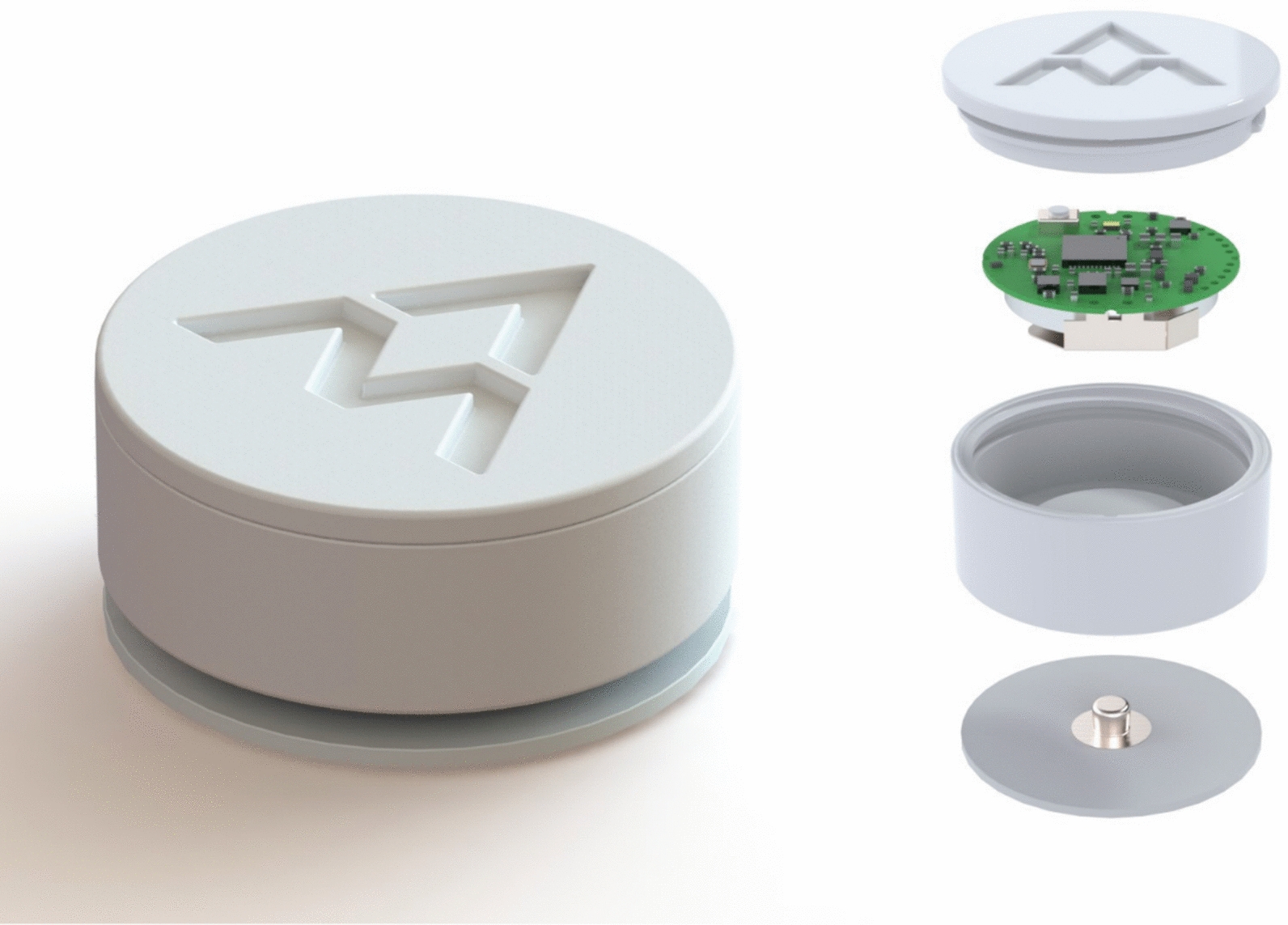
Appearance of the PneumoWave biosensor. The sensor can be seen on the left. On the right is the inside view of the biosensor, showing from top to bottom the housing lid, the accelerometer sensor, the sensor housing, and the ECG electrode used to mount the sensor onto the chest

The PneumoWave DC is a wearable biosensor that measures physical activity, consciousness, and respiration. The sensor attaches to its wearer’s chest using an ECG electrode and communicates with a gateway device (a “hub”) using Bluetooth to passively record respiration when within range of 10m^2^. Its functionality aims to alert bystanders or first responders to a patient’s respiratory issues [12]. Figure 1 depicts the appearance of the biosensor.

Participants were provided a sensor and hub to passively monitor their respiratory patterns, over a four-week period. and returned to the harm reduction service weekly for a total of five study visits, during which batteries were changed, and participant consent was re-confirmed.

The intervention involved this wearable respiratory biosensor designed to passively monitor breathing patterns associated with opioid overdose. Participants wore the device on their torso, below the clavicle, where it continuously recorded respiratory activity. The sensor sent data to a gateway device plugged into the wall when connected through Bluetooth, the range of the device being 10m. The device itself did not provide active monitoring, real-time alerts, or automated emergency responses during the study period.

Respiratory data collected by the device were used for research and feasibility purposes rather than to trigger live responses. Participants were informed that the biosensor would not initiate emergency services or contact responders in the event of respiratory depression.

Professional stakeholders were engaged to explore perceptions of how such a device could be implemented within existing services if active monitoring or response pathways were introduced in the future. Stakeholder discussions therefore focused on anticipated processes, responsibilities, and system requirements rather than on operational experiences during the study.

The primary aim of the study was to assess the acceptability of passive respiratory monitoring among PWUD and to explore perceived facilitators and barriers to potential future implementation of an actively monitored version of the intervention.

### Participants and sampling

Specialist nurses recruited participants from a needle and syringe provision (NSP) and harm reduction service in the city centre of Dundee, operated by a third sector organisation (Hillcrest Futures). The service has a long history of involvement in research studies conducted by the University of Dundee, and its user base are familiar with study participation. Inclusion criteria for recruitment of participants were current self-reported use of illicit drugs, attendance of harm reduction/opioid agonist therapy (OAT) clinics within the NHS Tayside area, and ability to adhere to study schedule.

Participants who consented to the larger trial, were asked if they would consent to be interviewed about their experiences with the sensor. They received verbal and written information, including a patient information leaflet, about the interview, which took place during their final trial visit. While there was no incentive for the interview itself, participants received £10 upon recruitment, £5 for subsequent visits, £20 for returning study equipment, and £3.80 per visit for travel expenses. Non-completing participants were offered a £10 incentive, along with travel costs for the interview.

Professional stakeholders were purposively sampled by the research team (KH and AR) to represent a broad range of views. All focus group participants had a prior professional relationship with the researchers.

Twenty participants who completed the RESCU study and one who did not, were interviewed. Eight focus groups were conducted with members of the Scottish Ambulance Service (SAS); the Dundee Non-Fatal Overdose Group (NFOD); support group members, staff of Scottish Families Affected by Alcohol and Drugs (SFAD); staff of Hillcrest Futures (the organisation hosting the NSP service); and with the harm reduction nursing team after the completion of RESCU, reflecting on their experiences conducting the study. An earlier formative focus group conducted by MC and AR at the NSP service with the harm reduction nursing team to facilitate study protocol creation was also included within the analysis. Table 1 (Appendix 1) contains the characteristics of study researchers.

### Recruitment and consent

All trial participant interviews were conducted as part of their final visit. Interview consent was obtained as part of consent procedures on an opt-in basis by research nurses based at the NSP service.

Professional services staff were approached for focus group participation through email and were consented during the recruitment process.

### Data collection

Semi-structured interviews were conducted in clinical and private rooms at the NSP service in Dundee’s city centre. Interviews were conducted by KH from February-May 2022. All interviews were audio recorded and lasted from 3 to 33 min, with the average interview lasting 16 min. This variance in interview length occurred due to participant availability and their willingness to share information with the researcher.

Focus groups with Scottish Families Affected by Drugs and Alcohol (SFAD), the Scottish Ambulance Service (SAS), the Tayside Non-Fatal Overdose (NFOD) group and one focus group with the harm reduction nursing team were conducted over Microsoft Teams^©^ video conferencing software, while focus groups with Hillcrest Futures and two harm reduction nursing staff focus groups were conducted in person as the service’s NSP service was the RESCU study site. Focus groups lasted an average of 45 min and were facilitated by AR, KH and MC, all of whom had prior experience conducting qualitative research with PWUD.

Interviews and focus groups were transcribed verbatim, noting pauses, laughter, and outside noises using square brackets. Transcripts were analysed using reflexive thematic analysis [13, 14]. This method was chosen due to its suitability for large data samples along with flexibility allowing for theme comparison and identification across datasets.

Transcripts were coded using line by line coding in NVivo 12.6 Plus analysis software by KH. Codes were collated into themes by hand, using Microsoft Excel for data management. The coding process was informed by field notes and analytical memos taken during data collection and analysis respectively. Themes generated by the analysis were reviewed by a researcher working on a study and a research nurse involved in study recruitment (AR and BS). On review, the perspective of a participant who did not complete the study and the nursing staff involved in trial recruitment were identified and a further interview and two focus groups with the nursing team involved in participant recruitment were conducted in February 2023.

COM-B and Normalisation Process Theory were used as analytic frameworks to organise and interpret findings, rather than as post hoc explanatory tools. Themes were interpreted using the conceptual framework of the COM-B model for behaviour change to identify capability, opportunity, and motivational factors for intervention uptake [15]. Normalization Process Theory (NPT) [16] was used to guide the identification of implementation factors expressed by focus group members. Themes were developed inductively and subsequently interpreted in relation to COM-B and NPT constructs. Some themes reflect multiple behavioural and implementation processes. Both approaches were helpful in identifying barriers and facilitators to wearing the device and implementing the intervention during our analysis.

After the analysis, a further review took place in which KH, AR and BS discussed the study findings, comparing them with existing studies and discussed the integration of the device into existing services.

### Reflexivity

KH was a PhD candidate with a psychology degree, who worked previously with vulnerable populations as a researcher. AR is a pharmacist and consultant in pharmaceutical public health, working as an academic (Teaching and Research), with an interest in addiction and recovery. BS is a specialist hepatology nurse, with a broad experience of working with PWUDs and undertaking research. MC was an MRes. Student with a psychology degree, who is now working as a resident medical practitioner.

## Results

**Fig. 2:**
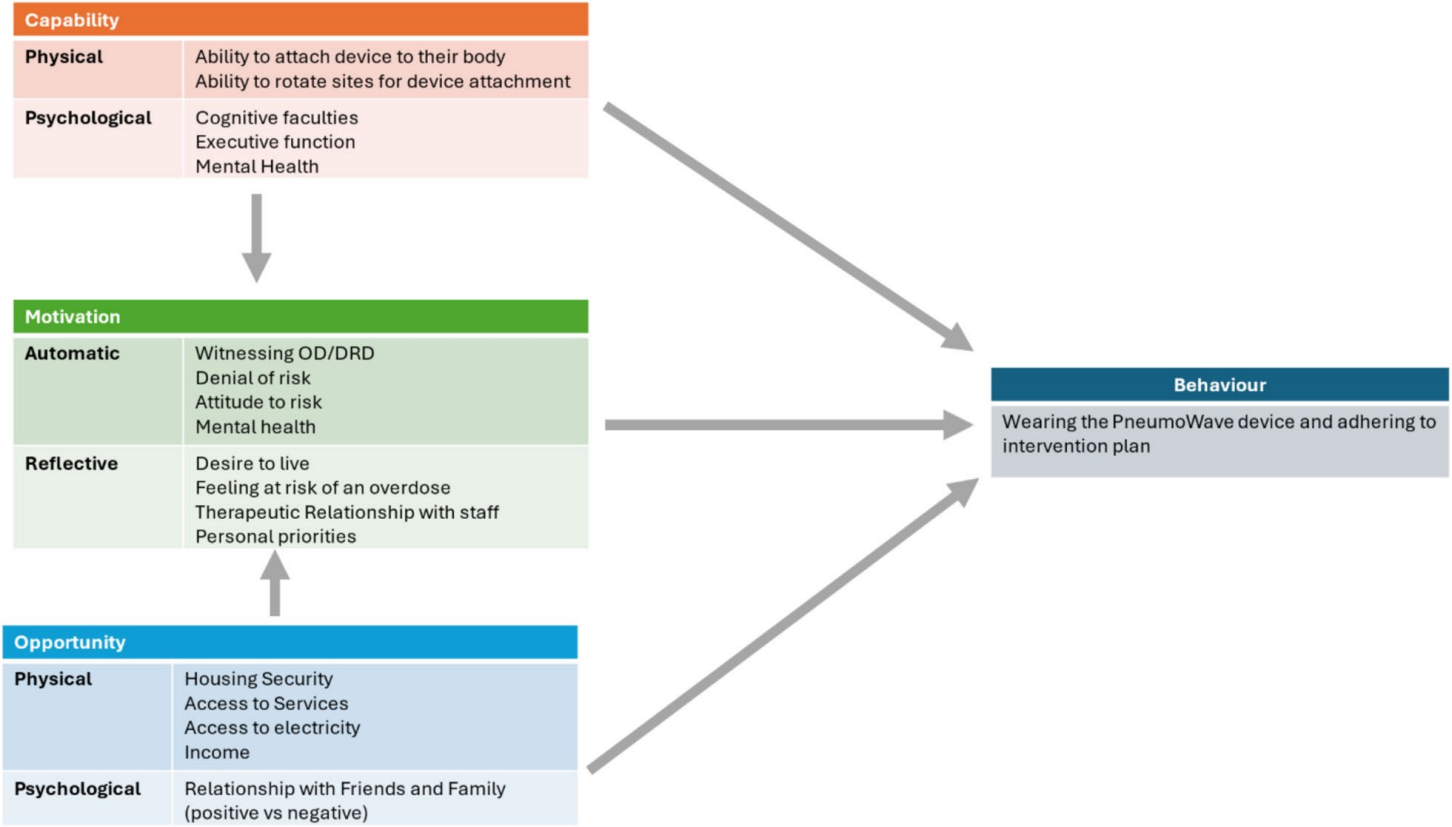
COM-B Model diagram of participant interviews

**Fig. 3:**
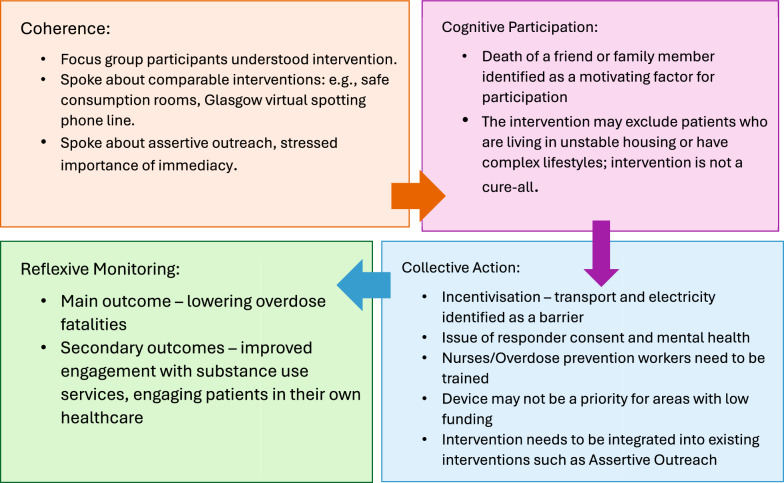
Professional stakeholder focus group normalisation process theory diagram

**Fig. 4:**
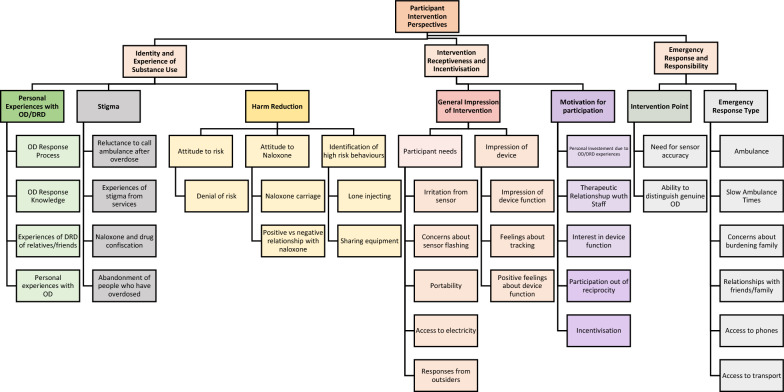
Participant interviews graphic

**Fig. 5:**
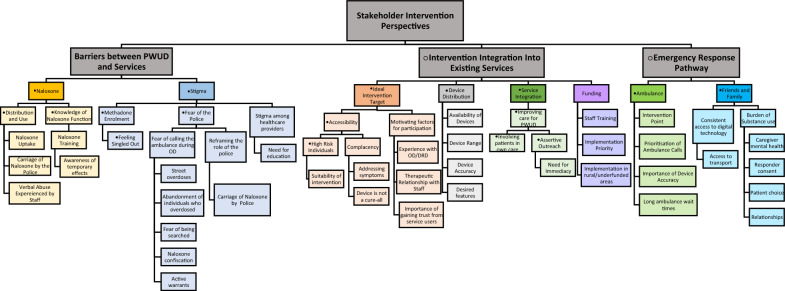
Stakeholder focus groups diagram

All semi-structured interview participants were individuals who had participated in RESCU, meeting the study’s inclusion criteria. 15 were male and six were female, with a mean age of 40. Tables 2 and 3 contain the characteristics of the interviewed participants and stakeholders (Appendix 1). Figures 4 and 5 (Appendix 2) contain the diagram of themes (*n* = 3 and 3) and subthemes (*n* = 7 and 7) of RESCU participant interviews and stakeholder focus groups respectively. Figure 2 displays the capability, opportunity and motivation factors contributing to the decision to wear the respiratory sensor as interpreted through the COM-B model for behaviour change. Figure 3 displays a diagram of the views expressed by professional stakeholders in focus groups as interpreted using Normalisation Process Theory. Tables 5 and 6 (Appendix 3) display the COM-B and NPT model tables displaying illustrating quotes. Table 7 (Appendix 4) displays a Scots to English dictionary.

PWUD and professional stakeholder findings are presented separately to reflect distinct experiential perspectives on the respiratory monitoring intervention. PWUD interviews focused on lived experiences of wearing the device and perceived acceptability, while professional stakeholder interviews explored anticipated implementation, responsibility, and system-level considerations. Where similar topics arose across both datasets, points of convergence and divergence are highlighted to demonstrate how perspectives were mutually reinforcing.

### PWUD interviews

#### Theme: identity and experience of drug use

Participants described knowledge about placement into the recovery position, administering naloxone and calling emergency services, which they often related to their experiences with overdose that reflexively motivated study participation.

Fifteen participants reported carrying naloxone administration, however two participants reported occasionally leaving their naloxone kits at home. One participant reported not carrying naloxone due to a lack of interest in aiding others, while another participant who had to exit the device study prematurely, had recently stopped injecting, and found the needle tempting.

Participants reported they would respond to an overdose, many said this was automatic. Participants identified social barriers to overdose response, reporting that many PWUD do not respond or choose to phone the ambulance anonymously and abandon the individual out of fear of prosecution, as police arrive with ambulances. These concerns echoed those raised by professional stakeholders, who similarly described apprehension about linking respiratory monitoring to emergency response due to the potential for police involvement and associated risks. Similarly, stakeholders also described the abandonment of people who had overdosed on the street. These observations represent social opportunity barriers.

Although concerns about police or emergency service involvement were raised, these did not appear to prevent engagement with the intervention among participants enrolled in the study. Instead, such concerns shaped how participants described preferred response pathways, where they said that despite there being high levels of mistrust of police who come with ambulance among PWUD, they preferred a response from an ambulance rather than friends or family who they believed to be less reliable.

“Because like, they’re like, immediately, they don’t think about things, they’re just like, maybe, if someone’s overdosing, they’ll go to the shop, get a bottle of juice, ken what I mean, then go and help.”

Interview Participant, Female, Aged 42 Years

Nine participants reported losing friends and family to drug overdoses, with seven commenting that these experiences motivated them to participate. Experiencing an overdose was a reflective motivation factor for participation, with participants stating their discovery was due to bystander presence or good fortune.

Participants spoke about their experiences with overdoses and acknowledged them as reflective motivation factors, recognising that they would likely have died if there was not somebody present to aid them during an overdose event.

"I OD’d one time and if it wasn’t for my mum coming to the, my house, I think I probably would have been dead, that kind of thing, because I mean, when my mum ended up banging on my door, she ended up kicking it in down there, ken, and she was kind of like [“Name!, Name!”], and my lips were blue and that. I was quite lucky not to die, or anything, ken.”

Interview Participant, Male, Age 32

Participants acknowledged risks of substance use, especially street benzodiazepines. This was likewise identified as a reflective motivation factor where participants felt at risk of an overdose which motivated them to participate in the study. However, conversely, there were automatic motivational factors where participants believed they were less at risk of an overdose compared to their peers. Participants stated they used clean injecting equipment which they did not share with others and believed they had safer injecting practices. The two participants who did not believe the intervention would benefit them identified that the intervention could benefit people who used drugs excessively.

I have, I have respect for what I’m taking […] which is something most people don’t have. […} Most people shouldn’t be let near drugs. […]

–Interview Participant, Male, Age 61

Participants’ decisions to take part in the study were shaped by reflections on personal risk and a desire to stay alive. Several participants described recognising how their substance use placed them at increased risk of overdose, particularly when using drugs alone at home, where naloxone could not be administered. Nine participants specifically identified lone injecting as a key risk factor, noting that previous overdoses had been survived through chance rather than safety. One participant described the death of his partner as a pivotal experience that motivated him to remain alive. These accounts reflect reflective motivation, as participants described deliberate assessments of risk and personal survival.

“Before, like, cause [name of previous partner] overdosed, living on the street, being a heroin addict for 20 year, or something like that, and then I lost my girlfriend last December. […] She was pregnant. Since that day, I’ve wanted to live.”

Interview Participant, Male, Age 45

“If you get people like that, ken, there’s no naloxone there, if they OD in the house, I’m never going to be able to get naloxone in to help them do it myself, don’t want, naloxone’s no going to be working, somebody else that could inject it into you, a device would come in handy for that obviously, for myself, for being in the house, like if I’m alone, like, ken. Uh, obviously, because you can’t inject yourself when you’re overdosing.”

Interview Participant, Male, Age 45

In addition to these reflective motivational factors, participants identified positive therapeutic relationships with NSP nursing staff as influential. Several participants described engaging with the intervention out of a sense of reciprocity, citing trust and encouragement from nursing staff as shaping their decision to participate.

“I enjoy [Specialist Nurse’s] company, he, he’s a thoroughgoing gentleman, he’s a first-class gentleman, I like [him] a lot, um, yeah. His enthusiasm is infectious, right, yeah. He’s a- you warm to him, you know what I mean, he’s very approachable, yeah, I like him a lot. I mean, if he asked me to do something, I would do it, you know, just because I like him.”

Male, Age 61

These motivations aligned with stakeholder expectations that the intervention could reduce overdose fatalities and strengthen engagement with services.

#### Theme: intervention receptiveness and incentivisation

Intervention receptiveness was discussed across both PWUD and professional stakeholder interviews, allowing for direct comparison of perspectives on acceptability and perceived value.

PWUD participants discussed receptiveness to the intervention primarily in relation to its use within their lives and issues related to it. Professional stakeholders described receptiveness in more operational terms, focusing on how the intervention might function in practice, including considerations related to implementation processes. Their accounts highlight how perceptions of the intervention differed across experiential and professional perspectives, while addressing related questions of feasibility and acceptability.

Multiple physical capability factors were identified. While thirteen participants stated that they found the device easy to wear, many participants struggled with the biosensor’s attachment. Fifteen participants found the electrodes hard to remove, and ten experienced skin irritation. Although patients were instructed to change electrodes and rotate sites, some did not, which was a concern for the nursing team due to the risk of skin problems. These usability issues were identified as a physical capability barrier to using the device. Some interview participants struggled with practical tasks, such as attaching the device or remembering to rotate sites, physical opportunity factors which indicated that ease of use is crucial for intervention adherence. Participants reported adhesive irritation as a significant barrier, suggesting a need for more user-friendly materials. Psychological capability factors, in this case mental and emotional factors, such as memory, attention, and mental health, also affected participation. Designing interventions that are simple and adaptable to different cognitive and emotional needs could improve both use and uptake.

“I just put it on and just forgot all about it, really. […] Except for taking that wee sticky bit, that was the only time I really noticed it. […] Do you know what I mean? But other than that, I didn’t even notice it was there, really.”

Interview participant, Male, Aged 32 years

“[The sensor] left a few marks when I, like obviously have not been washing daily, like you know it’s hard when you’re in addiction, like, to wash daily.”

Psychological capability factors which influenced participants’ ability to wear the device were the participants’ cognitive faculties, executive functioning, and mental health, often related to deprivation, which were identified by the focus group members. Focus group participants noted that participants would need to have the psychological capability to maintain their device.

Drug using and deprivation often very often go hand in hand. You know, if life is feels terrible. The last thing you want to do is face it in many cases. So, using drugs for that brief period of time, you can forget all about your troubles in your moves."

Male Participant, Nursing Team Focus Group

“…Everything changes from a day-to-day, but I, I think you’ve got to kind of put the onus on to the person as to whether they would be able to run with the schedule as such.”

Female Participant, Nursing Team Focus Group

While all interviewed participants were able to wear the device, six participants erroneously believed the device to be measuring heart rate or actively aiding respiration, likewise reflecting psychological capability factors.

Nine participants described the sensor’s light as intrusive in ways that affected their ability to tolerate and continue using the device. One participant with prior police involvement reported mistaking the sensor’s flashing for a police vehicle when waking during the night, which caused distress and disrupted sleep. Others described the visibility of the sensor light beneath clothing as leading to repeated questioning or confrontations, which heightened anxiety and made it difficult to manage use of the device in everyday settings. These experiences indicate that the intrusiveness of the sensor light compromised several participants’ psychological capability to comfortably engage with parts of the intervention.

[…]The only thing, the thing that really badgers is the light, especially at night, ken what I mean, especially if you’re getting a blue light, eh, ‘cause the first couple of days, aye, I kept forgetting that it was blue-so I’d wake up and I thought there was an ambulance or the police car outside or something, aye, cause there’d be like blue flashing lights and then I’d realise it was me, so I looked down on my chest, eh. That’s the only thing that sort of puts you off, eh.

Interview Participant, Male, aged 36 years.

Signal loss was reported by ten participants who reported losing signal within the same room, indicating device reliability issues that constrained consistent use. Thick walls in tenement flats therefore demonstrated a lack of physical opportunity to engage fully in the study.

Two participants reported benefits from study participation. One participant with a history of anxiety and depression felt enabled to leave the house indicating enhanced confidence and cognitive strategies to engage in daily activities, while another participant reported his drug diary, which participants kept as part of the main study to clarify respiratory patterns, alerted him to dangerous drug combinations that he consumed, showing cognitive engagement as psychological capability with the device and ability to interpret data. Six participants promoted the use of the device within their communities, indicating perceived value and psychological capability to advocate for its use.

Participants described how positive reactions from their social support system created a supportive social context for wearing the device in everyday settings. Several participants explained that favourable responses from those around them made it easier to explain the intervention and manage device use in social situations. Participants who expressed enthusiasm for the intervention noted that supportive responses from friends and family increased their confidence in navigating conversations and situations related to device use, facilitating continued engagement. These accounts display the role of social opportunity in supporting engagement with the intervention, alongside participants’ capacity to manage device use in social contexts.

Eighteen participants demonstrated psychological capability by articulating an understanding of their own overdose risk and how the respiratory monitoring device could function as a safety measure. Participants identified factors that placed them at increased risk of drug-related death, such as injecting alone, and described the device as a means of compensating for these risks by providing a potential safety net. Participants’ willingness to wear the device if it were to become standard practice reflected their capacity to recognise risk and engage with the intervention as a protective strategy for themselves and others. While these accounts also reflect motivational factors, participants emphasised understanding and risk recognition in explaining their engagement with the intervention.

“[…] not saying that I would have the free range to take any drugs that I want, but at least I knew, if I accidentally overdosed, like, somebody would be contacted, cause, as I say, I’m a person who decides the main company, a lot of the time. […] I rarely sit and do drugs with other people, eh, ken.”

Interview Participant— Male, Aged 36 years

Limited awareness of personal overdose risk reduced some participants’ ability to engage with the intervention. Two participants were indifferent to the intervention, believing they were not at overdose risk, an automatic motivation factor.

Participants’ ability to engage with the intervention was supported by access to stable housing, electricity, and transportation, highlighting how physical opportunity factors can enhance or constrain individual capability. Incentivization helped overcome barriers when these resources were lacking, enabling participation.

“Just to help us- we needs money, to get the electricity and food and stuff for us, so that was the positive for me...I needed that money, like I can’t get to the chemist sometimes, and I was getting the bus fares and that so that I could get to the chemist, so that was the positive for me.”

Interview Participant—Male, Aged 40

Participants showed psychological capability factors in identifying potential patients who would be able to benefit from the study. People engaging in high-risk behaviours, people who use benzodiazepines, and people with respiratory or heart issues were identified as ideal intervention recipients.

#### Theme: emergency response and responsibility

Distance from health services in Dundee was identified as a challenge, reflecting local health inequity. A psychological opportunity factor that was identified by the participants was the support of friends and family, as family and friends could provide valuable support, but for those hiding drug use, stigma and secrecy limited these networks.

Participants’ ability to use the intervention was shaped by both their environment and social context. The physical opportunity aspect of a non-intrusive, passive monitoring intervention made engagement easier. There was a desire for the device to have the ability to accurately distinguish overdoses from irregular breathing to create conditions where participants could engage with the intervention safely and with confidence.

“Not too sensitive because I’m, I’m a person with breathing problems anyway, so it might confuse my normal breathing problems with going over. Yeah, so I would say too sens- not, not too sensitive.”

Interview Participant, Male, Age 43

While dissatisfaction with emergency services was raised in nine interviews and in several stakeholder focus groups, ambulance attendance was the preferred method for most participants. Participants reported a lack of social opportunity, as they believed their friends or family were likely to be unreliable or unavailable due to a lack of a strong relationship, or due to keeping their substance use a secret. Participants stated friends and relatives may not have transport to facilitate a timely response, and an ambulance would need to be called regardless, resulting in wasted time.

“[…] friends and family, they can only do so much, that’s what they’d probably end up doing [calling the ambulance], they can do more, they’d go out and that’s just wasting more time and by the time they’d phoned, just, I’d say emergency services, the ambulance, straight away.”

Interview Participant, Male, Aged 35 years

These preferences echoed concerns raised by professional stakeholders regarding responder responsibility and capacity, highlighting shared uncertainty around appropriate emergency response pathways.

### Professional, family and healthcare stakeholder focus groups

#### Theme: barriers between PWUD and services

Stakeholders understood the purpose of the device being a respiratory monitoring device which would trigger during episodes of respiratory depression caused by drug use. However, as a form of cognitive participation, stakeholders noted fear of the police hindered PWUD from calling emergency services, a key part of the future intervention, due to risk of arrest and drug seizure fears, as police arrive with emergency services in Scotland. Nursing team members recounted narratives of overdose victims being moved to public spaces to avoid accommodation searching. Stakeholders noted that the recent carriage of naloxone by police officers had helped with gaining acceptance, but poor perceptions of emergency services remained a barrier. Focus group participants reported occasional negative or stigmatizing perceptions of police conduct by PWUD.

“You’re basically put out into a close or communal area, or somewhere that’s anonymous and then and then Joe Public phones an ambulance.”

-Focus Group participant, Harm Reduction Nursing Team

[On naloxone carriage by the police] I don’t think there’s any concerns about it at all. I think people are really quite happy and, and are promoting it quite well. So it’s been accepted pretty well.

Female Focus Group Participant, Non-Fatal Overdose Group

A fear of the police was also noted in participant interviews, but most of the participants stated that they preferred an ambulance response due to a lack of reliability or relationship with friends and family.

Stakeholders recognized stigma as a barrier for PWUD accessing services which they believed could be a potential problem for the distribution of this intervention. Parents in one of the focus groups mentioned that their children wanted opioid agonist therapy but disliked their pharmacy appointments due to feeling stigmatised. One focus group participant suggested reframing the police’s role as supportive rather than punitive. The nursing team noted that educating the public about substance use was a key way of addressing stigma as a point of collective action.

"Maybe changing their narrative and telling people why they’re doing that, like cause I think that people are just like, they’re taking my drugs, it’s all a criminal thing, but if it’s maybe made more that it’s for their own safety as well, changing the narrative might help"

Female Focus Group- Harm Reduction Support Staff

Stakeholders noted that naloxone distribution was high, but expressed concern about patient knowledge, reporting unawareness of its temporary effects and the need to call an ambulance after administration. This was notable part of cognitive participation as the future intervention would involve patients being administered naloxone after an alert would be made. Participants stated some PWUD viewed naloxone administration negatively due to causing immediate withdrawal from the injected drug, commenting verbal abuse received by first responders could negatively affect intervention effectiveness.

It was quite funny ‘cause we’ve had one guy who had t-who had gone over and beforehand he was saying “I don’t want naloxone”, we’re saying “look, if you go over, we’re going to have to give it” So he went over, so we gave him naloxone, and when he came around, we’re saying “look, this is just temporary, so, you know because you’ve had naloxone, but you need to not be going and using” and he’s saying “ I don’t want that, I’ll get a letter from my lawyer, [changes to a gentle voice] can I have 20 orange pins please?”

Female Focus Group Participant- Harm Reduction Support Staff

Focus group participants from the third sector organisation where the intervention study was carried out linked willingness to wear the device to collective experiences of overdose and concern for peers, highlighting how social relationships may foster cognitive participation in the intervention.

“I mean the people that are coming in here, pretty much all of them will know people that have passed through an overdose, so to avoid it happening to them or any more of their mates, pals, whatever, I think they’d be quite keen to wear that.”

Female Focus Group Participant, Hillcrest Futures

#### Theme: emergency response pathway

When identifying how the intervention should function in practice as a form of cognitive participation and collective action, parent support group members wished to be first responders, but other focus groups found issues with responder consent, access to transport and follow-up of responders, stating family responders may initially respond, but not for repeated alerts that could cause distress and add to psychological burden.

Focus group members emphasized that difficult relationships between PWUD and their families were a potential barrier to family members acting as first responders in collective action. Apart from relationship breakdown between service users and their families, harm reduction support staff noted that many service users do not admit to using drugs to their family, therefore using family members as first responders may not be possible for all participants. This was a sentiment noted in participant interviews.

While PWUD discussed nominated emergency responders primarily in terms of practical support and safety, professional stakeholders highlighted the emotional labour and boundary work involved for those taking on responder roles.

“…It would have to be someone that’s reliable, a lot of people don’t, don’t admit to using, so a parent or an, you know, maybe would not be an option, because parents maybe don’t know about their usage…I think that would be quite a difficult one.”

Female Focus Group Member, Harm Reduction Support Staff

Reliable transportation to reach rural patients and limited-service capacities in rural areas were also identified as challenges in implementing the intervention in terms of collective action. Harm reduction and nursing team focus groups reported slow ambulance response times, reporting that an ambulance could take up to 45 min to arrive at an overdose. Harm reduction staff reported that they needed to conceal nurse presence on site to prevent the ambulance service from taking longer to dispatch an ambulance due to a medical professional being on site. Harm reduction support staff also recalled ambulance dispatch being unfamiliar with naloxone, which could complicate the intervention process after being disseminated.

Research nurses involved in study recruitment recommended that patients should choose their preferred response method, as patient circumstances will differ, and some may prefer a response from their family. Supporting collective action, Scottish Ambulance Service members stressed the requirement for device accuracy as high call volumes cause service pressures and false alarms waste time and make patients less likely to use the device.

[…] How does it differentiate between somebody enjoying that hit, if you will, and going into overdose because for somebody who uses drugs, that will be a really important factor if they are going to wear that that well, if I’m gonna wear this in every time, you know, and I’m gouching. Does that automatically mean that somebody’s going to be, you know, kind of signaled?

-Female Focus Group Participant, Scottish Ambulance Service

#### Theme: intervention integration into existing services

Factors of collective action identified to promote equity of opportunity included secure housing, access to services, and appropriate incentivization, which were also highlighted in participant interviews as opportunity factors. Further areas of collective action which were identified by the stakeholders were training of staff involved in distributing the intervention and consistent access to devices, attrition of the latter being an issue during the quantitative study trial as participants lost their devices. Participants stated that areas with low funding may not prioritise this intervention due to lack of staff availability and due to resource implications.

“So again, if we’re talking about devices which are quite expensive however, then gonna look at that funding. Who’s going to get them? Is it going to be people that the Non-Fatal Overdose teams have already picked up or if we were going to get early intervention to try and prevent, there needs to be some kinda, say, set criteria I would say something so that there’s kinda parity of, of kind of, support across Scotland and it doesn’t become a post code lottery because one ADP area’s got a bit more funding and they’re all for it and other areas don’t get it because they don’t see as a priority.”

Female focus group member - Scottish Families Affected by Alcohol and Drugs (Staff)

Individuals at a high risk of overdose were identified as ideal intervention recipients. Hostels were mentioned as possible intervention settings due to staff’s ability to assume first responder roles. The intervention was identified as a method of retention in services. Nursing staff stressed that the device should not be used punitively (e.g. to monitor substance use by services). As a form of reflexive monitoring, nursing staff enthusiastically identified that the device had the potential to save lives of both committed patients and patients who lived more unpredictable lives, although they questioned if the intervention would benefit them equally.

“I think for specific individuals it would be absolutely perfect for them. Those who have the commitment to wearing a device such as this every day and who are willing to charge it up and have the commitment to wearing it. For them individuals, it’ll be absolutely spot on and it’ll be a game changer. And in terms of reducing drug related death, given that we do have a crisis for those who are a little bit more chaotic. I don’t know whether they will have the, the wherewithal to just to commit to that, but in terms of what it can deliver in theory, I think it’s absolutely spot on if it could trigger a next of kin or if it could trigger an ambulance from, from being called for an intervention. And in theory it’s absolutely it’s spot on.”

Female Focus Group Participant, Nursing Team

Furthermore, as part of reflexive monitoring, harm reduction nurses commented that the device had the secondary outcome of having potential to support PWUD in taking charge of their own health and to remain in contact with healthcare services as an ethos of harm reduction, referencing other healthcare trials that had used this approach.

“Nurses are not here to judge and tell you what to do, but here to advise you on the safest way for you to do whatever. [To] allow you to make an informed choice about your, about your own health care, because it does belong to you. It’s about giving you ownership.”

-Male Focus Group participant, Harm Reduction Nursing Team

As factors of cognitive participation and collective action, participants stressed the importance of immediacy in drug services contact, as service users did not recall their physical and mental state later after overdosing. Harm Reduction nurses stated that intervention uptake required a low threshold of entry and to distribute devices from areas where PWUD are comfortable and have a positive therapeutic relationship with staff, which was likewise identified in participant interviews as an important motivating factor.

[…] The set of outreach teams are out that afternoon trying to make contact with that individual because all the research tells us that if you could make contact with an individual in a shorter time as possible, post this event, the likely to be a bit more reflective and they’ll remember what happens if you do it, you know, three weeks or four weeks down the line or even a week down the line of kind of forgotten about what happened. And it’s not important to them anymore.

Female focus group participant, Non-Fatal Overdose Group

Stakeholders from the Scottish Ambulance Service noted that individuals with unstable housing or complex daily routines may be excluded from the intervention because they may struggle to follow the treatment plan or keep the device safe, reflecting a key cognitive participation challenge.

These stakeholder concerns reflected practical challenges and physical opportunity factors described by PWUD, including unstable housing, limited access to electricity, and difficulties maintaining the device in everyday settings. However, as part of reflexive monitoring, a parent support focus group member noted that this type of device could support individuals who are awaiting rehabilitation or a opioid agonist therapy prescription.

“I suppose whenever we think about new interventions, one of the things that always comes to mind for me is well, who are you talking about? Because often who gets left out is the individuals who are not attached to treatment services but who still don’t want to die from an overdose. And of course, oftentimes that then brings in other questions about how reliable it would be. So for example, maybe an individual who’s homeless not engaging with services, how likely are they to be able to safeguard their things, and how replaceable would something like this be? You know, so that we’re not thinking about punitive exiting of like the, the research because you’ve not been able to safeguard your equipment and things like that. And so these are the kind of things that floated around my head just in terms of inclusion and accessibility.”

Female Focus Group Participant, Scottish Ambulance Service

[…] If they’re engaging with that while they’re waiting to either get into rehab, just to be put on a prescription or whatever it may be […] one size doesnae fit all, and if that device is there as a coping mechanism ‘til they decide to go on with their treatment or their prescription or whatever, it could be a safety measure for them as well.”

Female Focus Group Participant, Parent Support Group

## Discussion

### Study findings

This study examined the acceptability, use, and implementation of a respiratory biosensor intervention designed to reduce the risk of fatal overdose among PWUD. Findings from interviews of PWUD participants who had participated in the main study, actively wearing the device and professional stakeholder focus groups who were presented the device to discuss its potential use, indicate that while the intervention was broadly perceived as acceptable and potentially lifesaving, its effective use and integration were shaped by interrelating individual and system-wide factors.

PWUD participants generally expressed strong motivation to engage with the intervention, largely driven by lived experiences of overdose, bereavement, and awareness of the risks associated with using drugs alone. A desire and a reflective motivation factor to remain alive and avoid being found at a stage where a medical intervention would not be successful featured prominently in accounts of why the intervention was seen as a potential solution to overdose deaths. However, this motivation was not uniform, and some participants expressed ambivalence or minimised personal risk, particularly when they believed that they were not at risk of an overdose due to being more experienced in using drugs than their peers.

Despite often high reflective motivation, sustained engagement with the intervention was constrained by capability-related factors. Participants described difficulties related to attaching and maintaining the device, physical discomfort and skin irritation. Psychological capability was also relevant, as some participants reported limited understanding of how the device functioned. These factors were also found in stakeholder interviews where it was acknowledged that some participants would not have the capacity to secure a device throughout the intervention, acknowledging that the intervention may inadvertently exclude some participants.

Opportunity-related factors strongly shaped whether and how the intervention could be used in practice. Housing instability, limited access to electricity, and the need to prioritise basic needs constrained engagement, while incentives were frequently used to support participation by offsetting these barriers and improved physical capability. Social support in this context was also found to be important. Trusting relationships with staff supported engagement, while concerns about stigma, device visibility, and unwanted attention influenced participants’ comfort with use.

Views on emergency response were complex. Many PWUD expressed a preference for professional medical assistance in the event of an overdose, while simultaneously describing fear or mistrust of police or emergency medical services based on past experiences.

Overall, professional stakeholders viewed the intervention as coherent, meaningful, and aligned with harm reduction goals. Stakeholders recognised its potential to address gaps in existing overdose prevention approaches by potentially providing a rapid response during periods of solitary drug use. Conversely, stakeholders raised concerns about the practical work required to implement the intervention, including responder burden, emotional labour from nominated responders, training needs, funding, and coordination with existing emergency and health services.

Stakeholders anticipated that the intervention could provide benefits beyond overdose prevention, including increased engagement with services and greater health awareness among PWUD. However, they emphasised that the sustainability and equity of the intervention would depend on system capacity and resource allocation, with risks of uneven access across settings, such as rural areas or areas with low funding.

Generally, the findings indicate that while motivation to prevent overdose and engage with the intervention was high among participants, successful and sustained use depended on addressing capability and opportunity constraints at the individual level and implementation challenges at the system level. Importantly, because all PWUD participants were enrolled in the intervention, perspectives of individuals who declined participation, potentially due to stronger mistrust of emergency services are likely underrepresented. As such, findings related to barriers and facilitators should be interpreted within this sampling context.

### Building on existing knowledge

Privacy and tracking had been identified as stressful for participants in previous literature [17, 18], however tracking was rarely mentioned as a concern in this study, with participants recognising that the device could provide protection when using drugs when injecting alone, a reflective motivation factor. It is important to note that the participants who agreed to participate in the study may have been participants who did not take issue with digital technology as participants with a fear of digital technology and tracking would not participate in a study of this type. Nursing staff noted that many PWUD including service users of the NSP service are distrustful of digital technology, and many PWUD are under skilled in its use, a factor of collective action which would need addressed. Stigma was also identified as a factor as it was identified that the device should not be used punitively.

Across both PWUD and professional stakeholder perspectives, findings converged around the importance of timely response and trust in those providing assistance. While PWUD emphasised safety, autonomy, and fears related to emergency response, stakeholders highlighted capacity, responsibility, and system constraints. Combined, these perspectives show how individual preferences and system-level considerations are mutually reinforcing in shaping the feasibility of the intervention.

Both participant groups discussed stigma experienced by PWUD in services, including feeling singled out when receiving OAT, or during ambulance calls. Reducing behavioral and attitudinal barriers and stigma underpins successful service implementation, as identified in recent studies [19, 20]. Stakeholders identified a need to change the police role narrative from hindering to helping, to decrease fear of the police. Participants identified a need to encourage PWUD to seek assistance during overdose events from the police and to understand that while police may confiscate drugs, this is not a punitive measure, but prevention of repeat overdoses. However, several narratives recorded that the mistrust of the police stemmed from a history of poor treatment of PWUD by the police, such as excessive or inappropriate searches. The nursing staff commented that police would on occasion be willing to search overdosing clients before their overdose was fully resolved. It seems likely that the cooperation between the police and overdose prevention initiatives should be undertaken in partnership. Recent research has made recommendations that overdose response needs to be reframed as a medical issue in order to prevent harmful behaviour from responding officers [21, 22]. This research, along with insights expressed by the participants in this study indicate that education and increasing awareness are pivotal to the success of overdose prevention initiatives.

While PWUD participants recognised that their substance use was putting them at risk of overdose, and many had experiences of overdose and drug-related death within their social circles, there appeared to be two coexisting views among participants. A correlation between the perception of overdose susceptibility and the use of protective behaviours has been documented in existing literature [23]. While participants acknowledged dangers of drug use, some were in denial of their own risk, believing they were more in control than others, corresponding to previous literature where knowledge of drug harm does not fully translate to accurate risk perception [24]. This indicates that there may be a disparity between the perception of overdose severity between patients and medical staff, leading to staff interfering during times that would not be perceived as serious by the by the patient, which may have implications for device uptake.

We also observed alienation from the PWUD community, as one participant noted that they would not be willing to provide aid in the event of an overdose. It is important to note that participants may have been inclined to respond in a socially acceptable way, and that the participants who had enrolled in the study may have been personally invested in overdose prevention and were therefore more likely to intervene.

Participants expressed a desire to be further engaged in their own healthcare and voiced an interest in the device being used in real-time monitoring. Engagement with healthcare services has been found to have a protective effect on health, with fewer people suffering from the harms of injecting drug use [25]. Harm reduction nurses stressed that it was important that service users gain a sense of ownership of their own health. Transformation of identity from a drug user to a person in recovery, has been identified as a factor in health-seeking behaviour [26]. This had also been evident in studies conducted at the NSP service prior to RESCU, such as the Eradicate-C trial in which participants who had been cured of hepatitis C reported changes in identity, including a decrease in injecting frequency [27].

Concerns were raised by focus group participants regarding responder consent and mental health. Although members of the parent support group expressed willingness to act as first responders, participants from other focus groups highlighted that frequent notifications, potentially multiple times per night, could have a serious impact on responders’ psychological wellbeing. Existing literature has documented the stress and burnout of overdose first responders [28–30]. Study participants shared perceived barriers for the use of friends and family as emergency responders, such as absence of stable relationships, reliability, and access to transport. Access to electricity and digital technology was a barrier in intervention implementation, as has been observed in other technology-based studies [31, 32]. Incentivisation was recommended to address this issue.

### Mechanisms and clinical implications

Motivation to participate in the study came from both automatic reactions and conscious reflection. Past overdose experiences, perceived risks, and mental health issues shaped automatic motivation. Reflective motivation included wanting to stay alive, fear of overdose, and supportive therapeutic relationships with staff. A striking finding of the study and reflective motivation factors were the importance of a good therapeutic relationship with harm-reduction centre staff for device acceptance. This indicates the importance of training for harm-reduction staff to build positive relationships between staff and clients to facilitate study participation. The barriers between PWUD and services were caused by perceived stigma among first responders. This indicates that building a positive therapeutic relationship with service users and the minimisation of stigma would improve engagement within patient services.

We identified incentivization as an opportunity factor and a factor of collective participation. Poverty, homelessness as well as exposure to poverty are common among people who use drugs [33, 34]. Experience of poverty in childhood has been associated with drug use disorders in adult life [35]. The fact that participants reported using their incentive payments to cover basic needs highlights the importance of the incentive as opportunity factors along with housing. This was corroborated upon data review, as participants in previous studies conducted in the service had reported spending their incentive payments on household items, such as a vacuum cleaner. Housing security, access to electricity and services were identified as social opportunity factors, as participants needed to have a power source from which to charge the device. These are vital factors to consider as in past studies, housing insecurity has been linked to an increase in fatal overdoses [36, 37]. PWUD who are living in unsecure housing have been found to be twelve times more likely to experience an overdose than the general population [38]. Signal loss was experienced by ten participants which was experienced even when the participants remained in the same room. Participants reported that the device’s signal did not reach the bathroom, which posed issues for participants who had plugged their gateway device in the bedroom, but used drugs in the bathroom, as the signal did not extend there. Therefore, for interventions utilising digital technology to be successful, contingencies need to be made to not inadvertently exclude this high-risk population group and the device needs to be mobile to reach participants when they are using drugs.

Multiple participants reported participating in the intervention out of reciprocity as they felt well-cared for by the injection equipment provision service staff and nursing team, therefore a positive therapeutic relationship with staff was identified as a factor of social opportunity, as was a supportive home environment. The importance of therapeutic relationships and their role in patient outcomes has been documented widely across literature [39, 40]. Nurse-led teams have been found to work as a bridge between the patients and access to wider support, therefore positive therapeutic relationships are important to retain engagement within services [41]. The findings of this study reiterate the importance of involving PWUD in intervention development. The direct input of this community ensures that the programs are tailored to address their needs and nurtures a sense of ownership of one’s own health. Therapeutic alliances between staff and service users have been previously found to contribute to service users decisions to pursue treatment [42].

Recently published studies report an increase of a public health approach to policing in Scotland, with police officers being issued with internasal naloxone [43]. Attitudes to this approach have been reported as generally positive, however, stigma is still present among the police force [44, 45]. Within this study, both participant groups expressed that fears about police persist among PWUD in Dundee, with participants reporting that people who have overdosed are being carried out of the house or abandoned after calling emergency services. Nonetheless, participants expressed that ambulance was the preferred emergency response method due to its reliability despite fears of prosecution, however both PWUD and focus group participants acknowledged that the ambulance service was stretched, resulting in slow response times. The harm reduction and nursing team focus groups believed this to partly be a result of a lack of dispatcher knowledge about naloxone’s temporary effects.

The automatic motivation factors involved were participants mental health, prior experience of an overdose, or witnessing an overdose or a drug related death, while reflexive motivation factors were participants’ attitudes to risk, including denial of risk, such as believing that their drug use was more under control than others’. Similar attitudes have also been present in previous studies, in which experienced PWUD who had been accessing services had expressed beliefs that they were at a lower risk of an overdose than their peers, and with people who had experienced an overdose feeling at risk [46–48].

Similarly to other studies focused on digital health interventions for PWUD, [49, 50], focus group participants noted that there were training needs associated with the intervention dissemination, which alongside costs associated with device provision would result the intervention to be of lesser priority in areas with low funding. Likewise with previous studies, service providers reported the intervention would not serve all patients, such as individuals unable to safeguard their device and attend services [50].

Participants identified lowering overdose fatalities as the primary outcome of this intervention in reflexive monitoring. Secondary outcomes were also proposed. The need to remain in touch with healthcare services for intervention monitoring was identified to potentially aid patients’ engagement with healthcare services. Interview participant responses identified the intervention has the potential to improve patient engagement in healthcare: a wrap-around supportive care package was necessary to maximise intervention utility. These findings corresponded to attitudes towards digital health technology in overdose prevention settings observed in literature, in which these novel interventions were received positively, but concerns were raised regarding funding in order for these interventions to be provided in the context of service delivery. For such an intervention to be successful, there is a need for consistent structural support [31, 50, 51].

The findings of this study indicate that a wearable biosensor is acceptable to PWUD and stakeholders and a viable intervention to explore in developing an alternative to SCRs for individuals who are unable or unwilling to attend physical SCRs, and for areas where physical SCRs are currently unavailable due to policy or funding issues. However, as has been noted in previous studies assessing digital health interventions, it is important to recognise that while digital health overdose prevention interventions show promise in saving lives, they address the most immediate consequences of drug use and not the underlying factors leading to drug use.

### Strengths and limitations

Focus group dynamics which were further aided by pre-existing histories with the interviewers facilitated a range of perspectives, while interviews with a non-completing participant and specialist nurses offered insights into study challenges. Participant recruitment by harm reduction nursing staff, who had long-standing therapeutic relationships with service users, enhanced the study findings.

Combining the COM-B and NPT frameworks provided a robust approach to understanding device acceptability and implementation, highlighting the interplay of capability, opportunity, motivation, and social context.

However, several limitations were identified. While participants were familiar with the interviewer from prior study visits, participants may not have been as open as they would be with a researcher with closer rapport.

Experiences with overdose and drug related death are deeply personal and traumatising. The emotional nature of the issues discussed within the interviews, along with participants’ challenging life circumstances, which often prevented them from remaining at the NSP service for extended periods, severely impacted interview length. One participant became very emotional when discussing her motivations to participate, as they involved the death of a close friend. This led to her asking if she could leave the interview. While her interview only lasted for three minutes, we chose to include the insights she had shared in her interview as she shared valuable information regarding motivations to participate in overdose prevention trials. One participant who was experiencing domestic violence could only be interviewed for five minutes, giving out all the necessary information rapidly so that she could return to her partner who was waiting for her outside of the service.

The exclusion of unstably housed individuals may limit the generalisability of findings, particularly regarding barriers such as access to electricity. Selection bias was a large factor, as the study primarily included participants engaged with NSP or OAT as this was one of the inclusion criteria of the main study which required that participants be receiving services in NHS Tayside for logistical reasons. This resulted in the recruitment of individuals whose lives were at a point of stability where they were able to consistently access NSPs and OAT programs. However, this resulted in underrepresenting unstably housed PWUD, and therefore the study does not include the most marginalized voices of PWUD. Furthermore, the study does not provide methods in which PWUD experiencing homelessness would be able to benefit in this intervention. We aimed to identify up to five participants who did not complete the full study to bridge this gap, however, we were only able to recruit one participant who, while not stably housed, had been undergoing OAT, therefore the most vulnerable PWUD were not represented in this study.

All PWUD participants in this study were enrolled in the intervention, meaning that individuals who declined participation, potentially due to stronger mistrust of emergency medical services or police, are likely underrepresented. As a result, findings related to fear of emergency response should be interpreted as reflecting concerns among those willing to engage, rather than as definitive barriers to uptake. Perspectives of individuals who were unwilling to participate may differ and warrant further investigation.

The study’s exclusion of unstably housed and homeless PWUD means their voices were not represented in this study. This is a significant limitation as these are the main individuals who face the most significant barriers and could benefit most from novel harm reduction strategies. The study also failed to explore how these individuals could access or continue using the intervention, which further limits the findings’ applicability to this vulnerable population.

While coding and manuscript review involved harm reduction specialists, the absence of peer researchers may have introduced undetected biases. Finally, COM-B’s simplifications and lack of specific evaluation tools required supplementation with NPT to provide a more comprehensive understanding. Broader evidence is needed to capture the perspectives of more vulnerable and underserved populations.

## Conclusion

Most participants received the intervention positively. RESCU participants found the device easy to use but found the attachment method intrusive. Stakeholders stressed the importance of appropriate overdose responders, integration among existing services and training distributors. Both RESCU participants and stakeholders emphasised the importance of positive therapeutic relationships between staff and service users.

The findings of this study indicate that a wearable device is acceptable to PWUD and stakeholders, meriting further research as an SCR supplement or alternative, but acknowledge the need to consider practicality and sustainable funding from within health services.

## Appendix 1: Researcher and participant characteristics

See Tables 1, 2, 3, 4.

**Table 1:** Researcher characteristics

Researcher’s Initial	Interviewer/Facilitator	Credentials	Occupation	Gender
KH	Interviewer/Facilitator	MSc	PhD Researcher	F
AR	Facilitator	PhD	Consultant in Public Health Pharmacy	M
MC	Facilitator	MSc(Res)	Clinical Researcher	F
BS	Research Nurse/Focus Group Participant	DipHE	Specialist Nurse	M

**Table 2:** Interview participant cohort characteristics

Variable		M	SD	n	%
*Gender*					
Men				15	71%
Women				6	29%
Non-binary				0	0%
Total				21	100%
Age					
		40	7.38		
*Living circumstances*					
		Homeless e.g. living on the streets		1	5%
		Living in temporary accommodation (e.g. shelter or hostel)		4	19%
		Staying with friends or family		4	19%
		Living in own home		12	57%
*Medical issues*					
		No Declared Medical Issues		13	62%
		Diabetes		0	0%
		Documented coronary heart disease (angina, CAD or previous MI)		1	5%
		Cerebrovascular disease (stroke or TIA)		0	
		Asthma		3	14%
		COPD		3	14%
		Sleep Apnoea		1	5%
		Pulmonary embolism		4	19%
		Overdose in last 6 months		1	5%
		Chronic anaemia		1	5%
		Epilepsy		1	5%
Mental health (in the past 6 months)					
		Depression		17	81%
		Anxiety		17	81%
		Suicide Attempt		4	19%
*On opioid agonist therapy*		Yes		17	81%
		No		4	19%

**Table 3:** Characteristics of focus group participants

Focus group	*n*	Mode
Scottish Ambulance Service	7	MS Teams
Hillcrest Futures Harm Reduction Staff	3	In person
Dundee Non-Fatal Overdose Group	7	MS Teams
Scottish Families Affected by Alcohol and Drugs (Staff)	10	MS Teams
Scottish Families Affected by Alcohol and Drugs (Parent Support Group)	6	MS Teams
Harm Reduction Nursing Team	6	In person
Harm Reduction Nursing Team	2	MS Teams

**Table 4:** Characteristics of full study participants

Variable	M	SD	n	%
*Gender*				
Men			48	68.57%
Women			21	30.00%
Non-binary			1	1.43%
Total			70	100%
*Age Distribution by Gender*				
Men			39.23	6.88
Women			37.00	5.36
Non-binary			31.00	N/A
Total			38.44	6.85
*Age Distribution*				
18–24			1	1.43%
25–34			19	27.14%
35–44			37	52.86%
45–54			12	17.14%
55–64			1	1.43%
65 +			0	0.00%
*Living Circumstances*				
Homeless e.g. living on the streets			2	2.86%
Living in temporary accommodation (e.g. shelter or hostel)			26	37.14%
Staying with friends or family			11	15.71%
Living in own home			31	44.29%
*Medical Issues*				
	No Declared Medical Issues		41	58.57%
	Diabetes		1	1.43%
	Documented coronary heart disease (angina, CAD or previous MI)		1	1.43%
	Cerebrovascular disease (stroke or TIA)		0	0.00%
	Asthma		12	17.14%
	COPD		6	8.57%
	Sleep Apnoea		3	4.29%
	Pulmonary embolism		7	10.00%
	Overdose in last 6 months		5	7.14%
	Other		6	8.57%
*Mental Health (in the past 6 months)*				
	Depression		62	88.57%
	Anxiety		63	90%

## Appendix 2

See Figs. 4, 5.

## Appendix 3: COM-B and NPT diagrams and quotes

See Tables 5, 6.

**Table 5:** COM-B items and supporting quotes

COM-B Component	COM-B factors	Supporting quote
Capability—Physical	Ability to attach device to their body	"Well, truthfully, I kept forgetting to- well I took the battery out when I’d shower, and I’ll try and wash around it, but it still gets a bit wet, so last week I came in here with sellotape stuck to it, but-just stuck to my body, I just stuck it on with the tape” [Shows sensor attached with duct tape]
		Female, Age 42
		“…this group of people you know, are are well, not much less, shall we say they’re just so chaotic that I don’t know how that will work, but I’m pleased to find out more and how it can be made workable for them.” Female Focus Group Participant, Non-Fatal Overdose Group
Capability—Physical	Ability to rotate sites for device attachment	“KH: did you have it stuck on the same place the whole time, or did you change the sides? Participant: no, I just kept it on the same bit. Yeah.” Male, Age 45
		“There’s a bit of a burn layer left if people are wearing it for more than four days or a week. I worry about that. If that was left on for a while, cause we have the very same analogy with dressings. So we put a dressing on and somebody doesn’t attend doesn’t move that dressing for over a week. Who’s responsible for that?”
		Male Participant, Nursing Team Focus Group
Capability—Psychological	Cognitive Faculties	“Drug using and deprivation often very often go hand in hand. You know, if life is feels terrible. The last thing you want to do is face it in many cases. So, using drugs for that brief period of time, you can forget all about your troubles in your moves.” Male Participant, Nursing Team Focus Group
		“…Everything changes from a day-to-day, but I I think you’ve got to kind of put the onus on to the person as to whether they would be able to run with the schedule as such.”
		Female Participant, Nursing Team Focus Group
Capability—Psychological	Executive Function	“That’s (the sensor sticker) been mostly there all the time, until I was going there and it fell off, and look at that, it’s been on the last minute, and it fell off”
		Male, Age 45
		“[The sensor] left a few marks when I, like obviously have not been washing daily, like you know it’s hard when you’re in addiction, like, to wash daily.”
		- Interview participant, female, aged 40 years
Capability—Psychological	Mental Health	“You know, there’s lots of mental health issues tied in with people who use drugs. We know there’s lots of abuse situations. We know there’s lots of, you know, there’s lots of histrionics which have led someone to that position.”
		Male Participant, Nursing Team Focus Group
		“I think they’ll need to be in a, a-in a place in their head that they don’t want to die”
		Female Participant, Scottish Families Affected by Alcohol and Drugs (Parent Support Group)
Motivation—Automatic	Witnessing or experiencing and OD/DRD	“First I freaked out, then I was slapping around the face, then I realised what had happened because the needle was still in him, so, I took the needle out, I wasn’t actually supposed to do that, I gave him naloxone and put him in the recovery position and phoned the ambulance.”
		Female, Age 27
		“I OD’d one time and if it wasn’t for my mum coming to the, my house, I think I probably would have been dead, that kind of thing, because I mean, when my mum ended up banging on my door, she ended up kicking it in down there, ken, and she was kind of like [“Name!, Name!”], and my lips were blue and that. I was quite lucky not to die, or anything, ken.”
		Male, Age 32
Motivation—Automatic	Denial of risk	“That’s why I ken my limit, I would never ever go over like, what I could handle, do you know what I mean? “
		Male, Age 36
		“Ken what I mean, I’m no stupid in that way, but most people just, might swallow the lot, you’re mental, one might be stronger than the other, ken.”
		Male, Age 35
Motivation—Automatic	Attitude to risk	“So I try, I try not to tak vallies if I’m injecting, kind of thing, do you know what I mean? I try not to tak vallies but sometimes I tak vallies with weed, when I smoke cannabis and that, I take them, so yeah, a multitude of things, do you know what I mean. […] I try no to mix the harder drugs with each other, ken, try and tak them, try and tak them on their own, kind of, do you know what I mean?”
		Male, Age 32
		I have, I have respect for what I’m taking […] which is something most people don’t have. […} Most people shouldn’t be let near drugs. […]
		-Male, Age 61
Motivation—Automatic	Mental health	[On study visits] “[I was] sort of pushing myself to get out of the house, because I’ve got anxiety and one tablet for anxiety and depression, so getting me out of the house, ken. I was pushing myself, ken”
		Male, Age 52
		“But that’s why I wanted to do it, I’ve got anxiety, depression and I use on my own as well, so that’s why this device, it would be handy for someone like me”
		Male, Age 36
Motivation—Reflective	Desire to live	“Before, like, cause [name of previous partner] overdosed, living on the street, being a heroin addict for 20 year, or something like that, and then I lost my girlfriend last December. […] She was pregnant. Since that day, I’ve wanted to live.”
		Male, Age 45
		“Basically, uh, but that’s, and it’s the fact that, as I said to you, I don’t want to [die]-I’ve got a sister to live for, a good family and that, I don’t want to…I want to have a life as well as just living.”
		Male, Age 45
Motivation—Reflective	Feeling at risk of an overdose	“‘I’ve been through intensive care and that before and I know, that, you know, that, like, if something did go wrong, it was only just by chance that, the last time, I was saved, I mean, it was just through a friend, I mean it was just happenstance that they came to my house that day, or y’know, I was in intensive care, and uh, a couple of hours, and I know, you know, they wouldn’t have been able to save me, so.”
		Male, Age 45
		“If you get people like that, ken, there’s no naloxone there, if they OD in the house, I’m never going to be able to get naloxone in to help them do it myself, don’t want, naloxone’s no going to be working, somebody else that could inject it into you, a device would come in handy for that obviously, for myself, for being in the house, like if I’m alone, like, ken. Uh, obviously, because you can’t inject yourself when you’re overdosing.”
		Male, Age 45
Motivation—Reflective	Therapeutic Relationship with staff	“I mean, my heart could stop one day, and [Specialist Nurse] told me, the, the future benefits of it. I mean, we can, uh, look where you are send an ambulance to you and that, so it’s just, it’s a good thing like
		Male, Age 43
		“I enjoy [Specialist Nurse’s] company, he, he’s a thoroughgoing gentleman, he’s a first-class gentleman, I like [him] a lot, um, yeah. His enthusiasm is infectious, right, yeah. He’s a- you warm to him, you know what I mean, he’s very approachable, yeah, I like him a lot. I mean, if he asked me to do something, I would do it, you know, just because I like him.”
		Male, Age 61
Motivation—Reflective	Personal priorities	“I would’ve loved to have something that tells me, the speed that my mum’s heart rate was going, ken, and if it alerted us earlier, it maybe could have saved her life, kind of thing, do you know what I mean?”
		Male, Age 32
		I’ve sort of realized myself that I want er, I want a better life
		Male, Age 45
Opportunity—Physical	Housing Security	“Yeah, I’m just tired from being awake for days, on the streets, y’know? But we’re staying with our friend, [friend’s name], all in the, [swallows], uh, [address], but he got the keys, see, on Friday, my friend [name], he got the keys on Friday, and because they don’t belong to me, I’ve no got around to sleeping.”
		Male, Age 45
		“At the time I was living in a homeless unit, got chucked out of there and ended up in another place. And then one morning I lost one monitor and then I lost the other bit, so I couldn’t keep participating in the [study]”
		Male, Age 36
Opportunity—Physical	Access to Services	‘You’re afraid to overdose at Whitfield [area in Dundee], you have more chance of dying if you overdose, because you’re furthest away from Ninewells Hospital, ken what I mean, but if you’re closer to Ninewells hospital, ken, you’ve more chance of [surviving]
		Male, Age 44
		“[On attending the NPS] You’re getting basically help, you’re not just coming in getting needles, basically, you’re getting help, and they’re looking after you more as well.”
		Female, Age 36
Opportunity—Physical	Access to electricity	“The only time it normally came off was in the bath or that, there’s been a couple of times when the electric’s went, but most of the time it’s collecting data, I would imagine.”
		Male, Age 35 “Aye, so not having a device and just this [sensor], yeah, yeah, but somebody who are on drugs and that, a lot of them no got, some of them don’t have electricity, I mean, I did, I got, that’s, if they have drugs and that, sometimes they forget to plug that device in, do you know what I mean, and if they’re out of electricity the device wouldn’t work. The wee sensor, the thingy, to contact the emergency services and get a person to help, eh.” Male, Age 36
Opportunity—Physical	Income	“I needed that money, like I can’t get to the chemist sometimes” Male, Age 40
		“I beg to make more money, I’ll try to make £25 for the gram.”
		Male, Age 45
Opportunity—Psychological	Relationship with Friends and Family (positive vs negative)	“[…]a lot of people have no got friends and families, gave up and ruined their friendships, ruined their families. Do you know what I mean?”
		Male, Age 52
		“[Family] could be scared of the consequences, or while the person is, while you’re lying, maybe they’d want to do you in.”
		Male, Age 32

**Table 6:** NPT Components and supporting quotes. The bold text respresents the definition of each phase

Normalisation process theory component	Meaning	Supporting quotes
Coherence	**The sense-making process where people understand and agree on what the new intervention entails, its value, and how it differs from existing practices**. Focus group participants understood intervention Participants spoke about comparable interventions: e.g., safe consumption rooms, Glasgow virtual spotting phone line Participants spoke about assertive outreach, stressed importance of immediacy	“What [I] heard actually of Glasgow, that, you can, there’s help line, when you’re on your own and injecting, or you’re using drugs you can phone a helpline and you can just you know, be with someone over the phone” Female Focus Group Participant, Hillcrest Futures “My worry about [the device is] that it might just be an alternative to drug consumption rooms or safe consumption rooms, which I think are something- I’ve always felt really passionate about” Female Focus Group Participant, Parent Support Group “[On the importance of immediacy of response]So we get the information [about the overdose] this morning, and the set of outreach teams are out that afternoon trying to make contact with that individual because all the research tells us that if you could make contact with an individual in a shorter time as possible, post this event, the likely to be a bit more reflective and they’ll remember what happens if you do it, you know, three weeks or four weeks down the line or even a week down the line of kind of forgotten about what happened. And it’s not important to them anymore.” Female Focus Group Member, Non-Fatal Overdose Group
Cognitive Participation	**The commitment and relational work individuals do to participate in and enact the new practice** Death of a friend or family member identified as a motivating factor for participation Some patients may not be ready for an intervention of this type; intervention is not a cure-all	“It’s quite an emotive and degree of the, it’s their incentive, it’s their motivation because the the, the appreciate, they’ve got peers that they’ve lost through drug death.” Male Focus Group Participant, Nursing Team I mean the people that are coming in here, pretty much all of them will know people that have passed through an overdose, so to avoid it happening to them or any more of their mates, pals, whatever, I think they’d be quite keen to wear that Female Focus Group Participant, Hillcrest Futures “I mean you know what it’s like with the chaotic people that we see, with that a phone number could last 24 h.” Female Focus Group Participant, Hillcrest Futures “This group of people you know, are are well, not much less, shall we say they’re they’re just so chaotic that I don’t know how that will work, but I’m pleased to find out more and and how it can be made workable for them Female Focus Group Member, Non-Fatal Overdose Group “I think we just need to remember that it’s not the be all and end all because no intervention is” Femaile Focus Group Member, Non-Fatal Overdose Group
Collective Action	**The operational work and effort undertaken to make the intervention happen and put it into practice** Incentivisation—transport and electricity identified as a barrier Issue of responder consent and mental health Nurses/Overdose prevention workers need to be trained Device may not be a priority for areas with low funding Intervention needs to be integrated into existing interventions such as Assertive Outreach	“I suppose electricity as well, quite often, they don’t always have electricity and that’s another big issue.” Female Focus Group Member, Nursing Team “I think if you incentivize it, that will bring [participation] closer.” Male Focus Group Member, Nursing Team I think it’s it’s a big ask for anybody to be part of that network, although I agree like in theory it’s like such a good idea and seems a bit no brainer, the more you explore it, the more questions you’ve got and I think that question of consent when people need to consent to be the people who respond and that needs to be alive, consent. “Do you agree tonight?” “Do you agree the next night” Female focus group member—Scottish Families Affected by Alcohol and Drugs (Staff) So if we’re trained, obviously, there shouldn’t be any issues, shouldn’t be any problem with that Female Focus Group Member, Hillcrest Futures Harm Reduction Staff “So again, if we’re talking about devices which are quite expensive however, then gonna look at that funding. Who’s going to get them? Is it going to be people that the Non Fatal Overdose teams have already picked up or if we were going to get early intervention to try and prevent, there needs to be some kinda, say, set criteria I would say something so that there’s kinda parity of of kind of, support across Scotland and it doesn’t become a post code lottery because one ADP area’s got a bit more funding and they’re all for it and other areas don’t get it because they don’t see as a priority.” Female focus group member—Scottish Families Affected by Alcohol and Drugs (Staff) “Yeah, absolutely, we’re saving lives. We’re keeping the person alive with the device but will be completely destroying the mental health and wellbeing of the family member.” Female focus group member—Scottish Families Affected by Alcohol and Drugs (Staff) “I can absolutely see the attraction of this for funders and for government, it’s like sexy, it’s shiny, it’s new, but actually not to over promise what it does and just to be really clear about what it does what it doesn’t do, and as [focus group member] said, it’s got to sit within that recovery-oriented system of care, not just as a kind of stand-alone, erm, standalone offer” Female focus group member—Scottish Families Affected by Alcohol and Drugs (Staff)
Reflexive Monitoring	**The formal and informal appraisal of the intervention’s impact, benefits, and costs by individuals and groups** Participants identified a main outcome of the information as lowering overdose fatalities Participants identified a secondary outcome, that the intervention could result in improved engagement with substance use services; engaging patients in their own healthcare	“There’s certain patients I’ve gotten in my head who this is perfect for, who gravitate to their own house, come in, use the exchange infrequently. Like there’s one person that is hardly ever in, I mean, but she stays at home and she’s got very poorly chest. She’s got very reduced mobility. I think she’s, since Day One, I’ve thought of certain patients.” Male Focus Group Participant, Nursing Team “I think for specific individuals it would be absolutely perfect for them. Those who have the commitment to wearing a device such as this every day and who are willing to charge it up and have the commitment to wearing it. For them individuals, it’ll be absolutely spot on and it’ll be a game changer. And in terms of reducing drug related death, given that we do have a crisis for those who are a little bit more chaotic. I don’t know whether they will have the the wherewithal to just to commit to that, but in terms of what it can deliver in theory, I think it’s absolutely spot on if it could trigger a next of kin or if it could trigger an ambulance from, from being called for an intervention. And in theory it’s absolutely it’s spot on.” Female Focus Group Participant, Nursing Team “it will bring out into the open even more, it will be another way of bringing out into the open that this is a medical health issue “ Female Focus Group Participant, SFAD Parent Support Group “[…]people then start to take ownership for their own drug use and their own health. So when they engage with the app, they can see well, actually, I can see for myself that my respiration felt below this level when I took that. That’s actually quite dangerous. And that’s just one of the things that can do could probably do a lot more” Female Focus Grouo Participant, Nursing Team “Treating substance use is more of like a chronic health issue. That project and very much so, which is recoverable through but not denying that. But at the same time it’s addressing the fact that at that moment in time about individual, it’s a chronic, it’s a chronic condition in which perhaps needs acute monitoring and especially if someone’s had high risk factors, Umm, nonfatal overdoses, recent liberation from prisons. You know, there will be other risk factors that puts people on the higher category of potentially being a list overdose and maybe, maybe the group that could target for inclusion in future trials” Male Focus Group Participant, Nursing Team

## Appendix 4: The scottish english dictionary

See Table 7.

**Table 7:** Scottish english dictionary

Scots	English
Aye	Yes
Ken	Know/Do you know?
Cannae	Cannot
Doesnae	Does not
Tak	Take
Vallies	Valium/Street Benzodiazepines

## Supplementary Information

Additional file1 (PDF 462 kb)

Additional file2 (DOCX 18 kb)

Additional file3 (DOCX 19 kb)

## Data Availability

Anonymised research data is available by reasonable request as per University of Dundee policy.
