# Supplementary material for: Acceptability and usability of a respiratory biosensor for drug overdose detection and first responder notification: a qualitative evaluation of perspectives from people who use drugs and wider stakeholders
Source: Harm Reduct J. 2026 May 21;23:119. doi: 10.1186/s12954-026-01452-8 (PMC13371256; doi:10.1186/s12954-026-01452-8)
Supplement: Supplementary file 2 — Additional file2 (DOCX 18 kb) [file 12954_2026_1452_MOESM2_ESM.docx]

### Table 2: Interview Participant Cohort Characteristics

| Variable |  | M | SD | n | % |
| --- | --- | --- | --- | --- | --- |
| Gender | |  | |  |  |
|  | Men |  | | 15 | 71% |
|  | Women |  | | 6 | 29% |
|  | Non-binary |  | | 0 | 0% |
|  | Total |  | | 21 | 100% |
|  | | | |  |  |
| Age |  |  |  |  |  |
|  |  | 40 | 7.38 |  |  |
|  | | | |  |  |
|  | |  | |  |  |
| Living Circumstances | | | |  |  |
|  | | Homeless e.g. living on the streets | | 1 | 5% |
|  | | Living in temporary accommodation (e.g. shelter or hostel) | | 4 | 19% |
|  | | Staying with friends or family | | 4 | 19% |
|  | | Living in own home | | 12 | 57% |
| Medical Issues | | | |  |  |
|  | |  | |  |  |
|  | | No Declared Medical Issues | | 13 | 62% |
|  | | Diabetes | | 0 | 0% |
|  | | Documented coronary heart disease (angina, CAD or previous MI) | | 1 | 5% |
|  | | Cerebrovascular disease (stroke or TIA) | | 0 |  |
|  | | Asthma | | 3 | 14% |
|  | | COPD | | 3 | 14% |
|  | | Sleep Apnoea | | 1 | 5% |
|  | | Pulmonary embolism | | 4 | 19% |
|  | | Overdose in last 6 months | | 1 | 5% |
|  | | Chronic anaemia | | 1 | 5% |
|  | | Epilepsy | | 1 | 5% |
| Mental Health (in the past 6 months) | | | |  |  |
|  | | Depression | | 17 | 81% |
|  | | Anxiety | | 17 | 81% |
|  | | Suicide Attempt | | 4 | 19% |
|  | |  | |  |  |
| On Opioid Substitution Therapy | | Yes | | 17 | 81% |
|  | | No | | 4 | 19% |
