# Supplementary material for: Acceptability and usability of a respiratory biosensor for drug overdose detection and first responder notification: a qualitative evaluation of perspectives from people who use drugs and wider stakeholders
Source: Harm Reduct J. 2026 May 21;23:119. doi: 10.1186/s12954-026-01452-8 (PMC13371256; doi:10.1186/s12954-026-01452-8)
Supplement: Supplementary file 3 — Additional file3 (DOCX 19 kb) [file 12954_2026_1452_MOESM3_ESM.docx]

### Table 4: Characteristics of the full RESCU study cohort

| **Variable** |  | **M** | **SD** | **n** | **%** |
| --- | --- | --- | --- | --- | --- |
| Gender | |  | |  |  |
|  | Men |  | | 48 | 68.57% |
|  | Women |  | | 21 | 30.00% |
|  | Non-binary |  | | 1 | 1.43% |
|  | Total |  | | 70 | 100% |
|  | | | |  |  |
| Age Distribution by Gender |  |  |  |  |  |
|  | |  |  |  |  |
|  | Men |  |  | 39.23 | 6.88 |
|  | Women |  |  | 37.00 | 5.36 |
|  | Non-binary |  |  | 31.00 | N/A |
|  | Total |  |  | 38.44 | 6.85 |
|  | | | |  |  |
| Age Distribution | | | |  |  |
| 18-24 | |  | | 1 | 1.43% |
| 25-34 | |  | | 19 | 27.14% |
| 35-44 | |  | | 37 | 52.86% |
| 45-54 | |  | | 12 | 17.14% |
| 55-64 | |  | | 1 | 1.43% |
| 65+ | |  | | 0 | 0.00% |
| Living Circumstances | | | |  |  |
| Homeless e.g living on the streets | |  | | 2 | 2.86% |
| Living in temporary accommodation (e.g. shelter or hostel) | |  | | 26 | 37.14% |
| Staying with friends or family | |  | | 11 | 15.71% |
| Living in own home | |  | | 31 | 44.29% |
| Medical Issues | | | |  |  |
|  | |  | |  |  |
|  | | No Declared Medical Issues | | 41 | 58.57% |
|  | | Diabetes | | 1 | 1.43% |
|  | | Documented coronary heart disease (angina, CAD or previous MI) | | 1 | 1.43% |
|  | | Cerebrovascular disease (stroke or TIA) | | 0 | 0.00% |
|  | | Asthma | | 12 | 17.14% |
|  | | COPD | | 6 | 8.57% |
|  | | Sleep Apnoea | | 3 | 4.29% |
|  | | Pulmonary embolism | | 7 | 10.00% |
|  | | Overdose in last 6 months | | 5 | 7.14% |
|  | | Other | | 6 | 8.57% |
|  | |  | |  |  |
| Mental Health (in the past 6 months) | | | |  |  |
|  | | Depression | | 62 | 88.57% |
|  | | Anxiety | | 63 | 90% |
